# Impact of RAAS Inhibitors on Clinical Outcome and Mortality in Patients With STEMI During the COVID-19 Era: A Multicenter Observational Study

**DOI:** 10.3389/fcvm.2021.792804

**Published:** 2021-12-24

**Authors:** Lucia Barbieri, Daniela Trabattoni, Giulio G. Stefanini, Enrico Vizzardi, Gabriele Tumminello, Emilio Assanelli, Marianna Adamo, Carlo A. Pivato, Giovanni Provenzale, Domitilla Gentile, Marco Metra, Stefano Carugo

**Affiliations:** ^1^Cardiology Unit, Fondazione IRCCS Ca' Granda Ospedale Maggiore Policlinico, Milan, Italy; ^2^Centro Cardiologico Monzino, IRCCS, Milan, Italy; ^3^Department of Biomedical Sciences, Humanitas University, Milan, Italy; ^4^IRCCS Humanitas Research Hospital, Milan, Italy; ^5^Cardiology, ASST Spedali Civili, Department of Medical and Surgical Specialties, Radiological Sciences, and Public Health, University of Brescia, Brescia, Italy

**Keywords:** STEMI patients, COVID-19, RAAS inhibitors, mortality, outcome

## Abstract

Conflicting results are available regarding the influence of ACEi/ARBs on the risk of COVID-19 infection, while less is known about their impact on the clinical outcome of patients with STEMI diagnosed with COVID-19. Our aim was to evaluate the impact of ACEi/ARBs therapy on in-hospital mortality and clinical outcomes of patients with STEMI during the COVID-19 pandemic. We retrospectively analyzed consecutive patients with STEMI hospitalized from February 20 to May 10, 2020 in four Hospitals in Lombardy. SARS-COV-2 diagnosis was performed by nasopharyngeal swab test. Procedural outcome, respiratory complications, and in-hospital mortality were reported. Univariate and multivariate analyses were performed by logistic regressions. Our population was represented by 182 patients with STEMI, 76.9% of which were males, and mean age was 67 ± 12.5. Hypertension was reported in 53.3%, and 29.1% was treated with ACEi/ARBs. COVID-19 diagnosis was confirmed in 17.1% of the patients. In-hospital mortality (13.2%) was significantly higher in patients with COVID-19 (31 vs. 10%, *p* = 0.003), even if ejection fraction [OR 0.93 (95% CI) 0.87–0.99; *p* = 0.03] and respiratory complications [OR 9.39 (95% CI) 1.91–45.9; *p* = 0.006] were the only two independent predictors. The incidence of COVID-19 infection was not influenced by ACEi/ARBs (16.5 in naïve vs. 18.8%) whose presence on admission did not correlate with respiratory complications or mortality both in the case of discontinuation and maintenance. In conclusion, in a high-risk population, such as that of patients with STEMI, the potential benefit of ACEi/ARB discontinuation in patients with COVID-19 is overcome by its detrimental effect. Intensive care, additional preventive respiratory investigations, regardless of swab test result, should be suggested for all patients admitted for STEMI during the pandemic.

## Introduction

Coronavirus disease-2019 (COVID-19) is a global pandemic that has affected more than 239,000,000 patients worldwide (WHO data report, October 14th, 2021). Italy was one of the most affected countries in Europe, especially in Lombardy. COVID-19 is caused by severe acute respiratory syndrome coronavirus 2 (SARS-CoV-2), which penetrates cells through the angiotensin-converting enzyme 2 receptor (ACE-2) (1). ACE-2 is largely expressed in the vascular endothelium and in the lungs (2). After ACE-2 was confirmed to be the SARS-CoV-2 receptor (3), it was supposed that treatment with angiotensin converting enzyme inhibitors (ACEis) and angiotensin receptor blockers (ARBs) would be harmful for COVID-19 patients. Few animal models have shown different results regarding the use of ARBs on ACE-2 with limited data in humans studying the effects of renin-angiotensin-aldosterone system (RAAS) inhibition on ACE-2 expression (4). There is a potential rather than harmful benefit in the use of ACE inhibitors. Meng et al. (5) suggested that the use of ACEi might be protective against respiratory complications. The binding of SARS-CoV-2 to ACE-2 exhausts ACE-2, with a consequent imbalance of the RAAS, which spirals into acute severe pneumonia. Blocking the RAAS by ACEi might reduce inflammation with potential reduction in mortality. Moreover, abrupt withdrawal of RAAS inhibitors in high-risk patients, like those affected by stage 3 hypertension, heart failure, or chronic coronary syndrome, may result in clinical instability and adverse clinical outcomes (6). The impact of RAAS inhibitors on clinical outcome has never been investigated on patients with COVID-19 presenting with ST-segment-elevation myocardial infarction (STEMI). Although it is well-known that all-cause mortality is reduced by 36% with an absolute reduction of 11.4% by the use of ACEi in post-infarct patients, their safety and effectiveness in patients with COVID-19 affected by acute myocardial infarction is unclear (7). The aim of this study was to evaluate the impact of RAAS-inhibitors on in-hospital mortality and clinical outcomes of patients with STEMI patients during the pandemic.

## Methods

We retrospectively included consecutive patients with STEMI who were hospitalized from February 20 to May 10, 2020 in four hospitals with 24/7 cath lab service in Lombardy. All clinical, demographic, and procedural characteristics were collected from a dedicated database. The study was performed in accordance with the Declaration of Helsinki. All the patients signed specific consent and disclosure for the use of personal data that were collected anonymously. The diagnosis of SARS-COV-2 was performed by nasopharyngeal swab test. Hypertension was defined as systolic ≥140 mmHg and/or diastolic blood pressure ≥90 mmHg or if on-treatment with antihypertensive medications (8). For patients on therapy with ACEi or ARBs, specific molecule and relative dose were reported, as well as discontinuation of such therapies after admission. For each patient procedural outcome, respiratory complications and in-hospital mortality were reported. Respiratory complication was defined as acute respiratory failure with a need for non-invasive and mechanical invasive ventilatory support. Ischemic stroke, non-fatal myocardial infarction (MI) and major bleeding, defined from types 3–5 according to Bleeding Academic Research Consortium (BARC) criteria, were identified as in-hospital adverse events (9). A statistical analysis was performed with the SPSS 23 statistical package. Continuous data were expressed as mean ± SD, and categorical data as percentage. For continuous and categorical variables, analysis of variance and chi-square test, respectively, were performed. Univariate and multivariate analyses were performed by logistic regressions, and a *p*-value < 0.05 was considered significant.

## Results

Our population was represented by 182 patients with STEMI, 76.9% were males with a mean age of 67.01 ± 12.5 years, and ranged from 40 to 92 years old. Baseline clinical and demographical characteristics are detailed in Table 1 for the whole population and divided in two subgroups according to COVID-19 infection. Hypertension was reported in 53.3% of cases treated with ACEi or ARBs in 18.7 and 10.4% respectively. The most used ACEi molecule was ramipril (61.7%), while olmesartan (26.3%) was for ARBs. ACEi's discontinuation was observed in 3/34 patients, due to hypotension and one of these patients resulted COVID-19 positive, while 9/19 patients in therapy with ARBs discontinued due to hypotension (4/19) or to ACEi switch (5/19). Regarding COVID-19 infection, a total of 169/182 patients were tested with nasopharyngeal swab test, with confirmed diagnosis in 17.1% of cases. Patients with COVID-19 showed higher baseline glycaemia on admission (151 ± 47 vs. 129 ± 51, *p* = 0.03), and lower rate of multivessel disease (21.4 vs. 52.9%, *p* = 0.002). Procedural and event characteristics are shown in Table 2. In the majority of cases STEMI diagnosis was assessed in a pre-hospital setting (51.6%), cardiogenic shock on admission was present in 8.8%, while 7.7% of the patients experienced out-of-hospital cardiac arrest. Anterior MI was the most represented (51.5%), and 12.6% of the patients needed intraaortic balloon pump support during primary PCI. Multivessel disease was detected in 47.8% of the cases, with complete revascularization in 22.9% during “index procedure” and in 42.5% staged before hospital discharge. In-hospital mortality was 13.2%, and in the multivariate analysis, ejection fraction [OR 0.93 (95% CI) 0.87–0.99; *p* = 0.03] and respiratory complications [OR 9.39 (95% CI) 1.91–45.9; *p* = 0.006] were the only two independent predictors. In our population, patients with defined diagnosis of COVID-19 showed higher incidence of respiratory complications and mortality rate (20.7 vs. 4.3% and 31 vs. 10%, *p* = 0.002 and *p* = 0.003 respectively) without significative increase in global in-hospital adverse events (34.5 vs. 19.3, *p* = ns). Nevertheless, the infection did not result as an independent predictor of mortality in both the multivariate and univariate analyses. The incidence of COVID-19 infection was not influenced by the presence of previous ACEi/ARBs therapy (16.5% in naïve patients vs. 18.8%; *p* = 0.73). We did not find any significant correlation between ACEi/ARBs therapy and respiratory complications [OR 1.08 (95% CI) 0.32–3.7; *p* = 0.89] or mortality [OR 0.45 (95% CI) 0.14–1.37; *p* = 0.15] in the whole population or in the COVID-19 subgroup [OR 2.83 (95% CI) 0.44–18.04; *p* = 0.27; OR 0.15 (95% CI) 0.01–1.46; *p* = 0.1; respiratory complications and mortality, respectively]. Among patients who discontinued ACEi/ARBs therapy, there was no more incidence of mortality (8.3 vs. 10%, *p* = 0.89) or respiratory complications (16.7 vs. 10%, *p* = 0.65).

**Table 1:** Baseline characteristics of the population.

**Clinical characteristics (*n*)**	**Total (182)**	**COVID – (153)**	**COVID + (29)**	***P***
Age (mean ± SD)	67.01 ± 12.5	66.3 ± 12.9	67.7 ± 10.5	Ns
Male gender % (*n*)	76.9 (140)	77.1 (118)	75.9 (22)	Ns
Diabetes % (*n*)	14.3 (26)	14.3 (24)	6.9 (2)	Ns
Hypertension % (*n*)	53.3 (97)	51.4 (79)	62.1 (18)	Ns
Family history of CAD % (*n*)	18.7 (34)	17.1 (26)	27.6 (8)	Ns
Active smoke % (*n*)	28.6 (52)	32.1 (48)	13.8 (4)	Ns
Previous smoke % (*n*)	4.4 (8)	2.9 (6)	6.9 (2)	Ns
Dyslipidemia % (*n*)	24.7 (45)	25.7 (40)	17.2 (5)	Ns
Ejection fraction (mean ± SD)	46.3 ± 10.5	46.8 ± 10.5	42.5 ± 9.2	Ns
Previous PCI % (*n*)	16.5 (30)	18.6 (26)	13.8 (4)	Ns
Previous MI % (*n*)	13.7 (25)	15.0 (21)	13.8 (4)	Ns
Previous CABG % (*n*)	1.6 (3)	2.1 (3)	0	Ns
Chronic kidney disease % (*n*)	17 (31)	17.1 (25)	20.7 (6)	Ns
COPD % (*n*)	10.4 (19)	8.6 (15)	13.8 (4)	Ns
COVID + % (*n*)	17.2 (29)	–	100 (29)	Na
**Biochemistry**				
Hemoglobin (g/dL) (mean ± SD)	13.5 ± 1.9	13.6 ± 1.8	12.9 ± 1.9	Ns
White Blood Cells (10∧3/uL) (mean ± SD)	11.3 ± 4.3	11.2 ± 4.0	12.5 ± 5.4	Ns
Lymphocytes (10∧3/uL) (mean ± SD)	2.2 ± 1.8	2.2 ± 1.8	1.7 ± 1.3	Ns
Platelet count (10∧3/uL) (mean ± SD)	243 ± 77	238 ± 68	280 ± 105	0.04
Creatinine (mg/dL) (mean ± SD)	1.04 ± 0.54	1.04 ± 0.57	1.09 ± 0.42	Ns
Creatinine peak (mg/dL) (mean ± SD)	1.39 ± 0.92	1.40 ± 0.99	1.43 ± 0.72	Ns
Creatinine Kinase MB (ug/L) (mean ± SD)	183 ± 316	201 ± 344	59 ± 59	Ns
Glycaemia (mg/dL) (mean ± SD)	134 ± 52	129 ± 51	151 ± 47	0.03
Reactive protein C (mg/dL) (mean ± SD)	32 ± 62	31 ± 61	43 ± 72	Ns
**Pharmacological therapy at admission**				
Acetylsalicylic acid % (*n*)	24.7 (45)	25.7 (38)	24.1 (7)	Ns
Vitamin K antagonists % (*n*)	1.1 (2)	2.2 (2)	0	Ns
Direct oral anticoagulants % (*n*)	3.3 (6)	3.3 (4)	12.5 (2)	Ns
Beta-blockers % (*n*)	24.2 (44)	25.0 (36)	27.6 (8)	Ns
Calcium channel blockers % (*n*)	15.4 (28)	17.1 (24)	13.8 (4)	Ns
Angiotensin converting enzyme inhibitors % (*n*)	18.7 (34)	18.6 (28)	20.7 (6)	Ns
Angiotensin receptor blockers % (*n*)	10.4 (19)	9.3 (16)	10.3 (3)	Ns
Statins % (*n*)	33 (60)	37.1 (53)	24.1 (7)	Ns
Aldosterone antagonists % (*n*)	8.8 (16)	9.3 (13)	10.3 (3)	Ns
Diuretics % (*n*)	27.5 (50)	27.9 (40)	34.5 (10)	Ns

*M-SD, mean-standard deviation; CAD, coronary artery disease; PCI, percutaneous coronary intervention; MI, myocardial infarction; CABG, coronary artery by-pass graft; COPD, chronic obstructive pulmonary disease*.

**Table 2:** Event characteristics.

**Site of diagnosis**	**Total (182)**	**COVID – (153)**	**COVID + (29)**	***P***
Pre hospital setting % (*n*)	51.6 (94)	52.9 (83)	37.9 (11)	Ns
Emergency department % (*n*)	41.2 (75)	40.7 (61)	48.3 (14)	
Spoke center % (*n*)	6.6 (12)	6.4 (9)	10.3 (3)	
Hub hospital department % (*n*)	0.5 (1)	0.0 (-)	3.4 (1)	
Out of hospital cardiac arrest % (*n*)	7.7 (14)	8.6 (13)	3.4 (1)	Ns
Killip class ≥3% (*n*)	11.5 (21)	9.8 (15)	22.2 (6)	Ns
Cardiogenic shock % (*n*)	8.8 (16)	7.1 (11)	17.2 (5)	Ns
**Procedural characteristics**				
***MI type***				
Anterior MI % (*n*)	51.5 (92)	49.3 (76)	55.2 (16)	Ns
Inferior MI % (*n*)	32.3 (57)	34.3 (49)	27.6 (8)	
Lateral MI % (*n*)	10.8 (18)	8.6 (16)	6.9 (2)	
Posterior MI % (*n*)	5.4 (8)	4.3 (7)	3.4 (1)	
Radial Approach % (*n*)	86.8 (158)	88.6 (137)	75.0 (21)	Ns
Multivessel disease % (*n*)	47.8 (87)	52.9 (81)	21.4 (6)	0.002
Left main % (*n*)	4.9 (9)	6.4 (9)	0	Ns
Left anteriore descending % (*n*)	62.1 (113)	61.4 (96)	60.7 (17)	Ns
Left circumflex % (*n*)	35.7 (65)	37.1 (59)	21.4 (6)	Ns
Right coronary artery % (*n*)	46.2 (84)	50.0 (74)	35.7 (10)	Ns
IABP % (*n*)	12.6 (23)	12.1 (18)	17.9 (5)	Ns
Final TIMI flow 3 % (*n*)	95.1 (175)	96.4 (147)	96.3 ()26	Ns
In hospital complete revascularization % (*n*)	42.5 (37)	40.5 (35)	40.0 (2)	Ns
Index procedure complete revascularization % (*n*)	22.9 (20)	23.3 (20)	0	Ns
**Main time components of STEMI care**				
Symptoms to first allarm (min) (mean ± SD)	437 ± 920	387 ± 87	83 ± 1486	Ns
Door to balloon (min) (mean ± SD)	57 ± 163	61 ± 183	37 ± 38	Ns
Total ischemic time (min) (mean ± SD)	514 ± 975	465 ± 71	899 ± 502	Ns
**In hospital outcome**				
In hospital adverse events % (*n*)	20.9 (38)	19.3 (28)	34.5 (10)	Ns
Respiratory complications % (*n*)	7.1 (13)	4.3 (7)	20.7 (6)	0.002
In hospital mortality % (*n*)	13.2 (24)	10.0 (15)	31.0 (9)	0.003

*IABP, intra aortic balloon pump; STEMI, ST segment elevation myocardial infarction*.

## Discussion

Among the patients with STEMI s during the COVID-19 pandemic, our study did not find any association between ACEi/ARBs use and mortality, and does not suggest the need to change our clinical practice. A report underlined the theoretical mechanism of action of ACEi and ARBs on RAAS and their potential influence on COVID-19 pathophysiology (6). The authors concluded the need for more data on this issue due to the potential positive effects of both discontinuation and maintenance of ACEs/ARBs during COVID-19 infection. The idea that ACE-2 receptor induction by ACEi/ARBs inhibition may be harmful during COVID-19 infection is based on animal models. Although no randomized trials or systematic analyses are available on the effects of RAAS blockers during the course of COVID-19 disease, and the European Society of Cardiology (ESC) and The American College of Cardiology (ACC) suggested physicians to continue the treatment in patients who are already on RAAS blockers. Nevertheless, it has also been recommended to individualize treatment decisions according to each patient's hemodynamic status and clinical presentation (10). The impact of arterial hypertension on cardiovascular disease risk is estimated to be up to 40% (11), which is reduced by the use of ACEI and ARB's. To date, it is still controversial, without any evidence, whether ACEI and ARB's could be harmful to patients with COVID-19. The high debate in social media, and popular and scientific journals forced major international societies to issue position statements. Fang et al. reported a possible impact of ACEI and ARBs on patients with COVID-19, which led to spread of alarm among physicians and patients (12). This finding was complicated by the evidence that arterial hypertension and diabetes mellitus are the highest co-morbidity of patients with COVID-19. In a case series of hypertensive patients from Wuhan, the absence of association between ACEi/ARBs and severity or mortality of COVID-19 was reported (13). Similar results were found in other studies from Italy (14) and North America (15, 16). ACEi/ARBs treatment is the cornerstone of cardiovascular therapy found to be effective in reducing death and cardiovascular end points in several settings. Early RAAS inhibition in patients with STEMI is safe and is associated with significant reduction in 30-day mortality (17). Clinical trials (18, 19), in the non-COVID-19 era, have shown that the discontinuation of ACEi/ARBs, especially after MI, correlates with worse prognosis. STEMI is a specific setting where hemodynamic impact plays a central role in the pathophysiology of the disease and may influence the underlying mechanism and prognosis (20). Li et al. since 2003 suggested that ACE-2 is a functional receptor of the SARS-CoV virus (21). Two years later, it was evidenced that virus–ACE-2 receptor binding plays a crucial role in cell penetration and consequent disease development (1). After the spread of COVID-19, several studies have confirmed that the SARS-CoV2 receptor-binding domain interacts with ACE-2 (22). On the other hand, ARBs might prevent inflammation of the lungs, as shown in animal models. Based on these data, reviews speculated that ARBs could be a possible treatment tool in patients with COVID-19 (23). A recent large observational study supports that, when clinically indicated, ACEi/ARB therapy should be continued in the setting of COVID-19 unless the patient is hemodynamically unstable (24). Even in this peculiar cardiovascular condition, with the co-presence of COVID-19 infection (25, 26), this study confirms that the use of ACEi/ARBs does not correlate with a worse in-hospital prognosis. Conversely, as the pre-COVID-19 era literature shows, untreated patients showed a doubled in-hospital mortality vs. patients receiving ACEi/ARBs, despite these data being not statistically significant. The multivariate analysis showed that ejection fraction and respiratory complications are the only variables correlating with in-hospital mortality. On the other hand, even if patients with COVID-19 had doubled mortality, the infection did not correlate in the multivariate analysis. The low negative predictive value of nasal swab test (27) may explain these findings. Therefore, regardless of swab test result, our results suggest the need for more attention to patient with STEMI and concomitant respiratory impairment, and an early preventive diagnostic and therapeutic approach. Contrary to the recent literature, in our study, patients treated with ACEi/ARBs did not show higher prevalence of COVID-19 infection, and therapy discontinuation did not influence both in-hospital mortality and respiratory complications. Therefore, the well-known beneficial effect of ACEi/s therapy in patients with STEMI is not balanced by the potential and not yet confirmed positive effect of discontinuation in the presence of COVID-19 infection.

## Limitations

The routine use of nasopharyngeal swab as the only screening test for COVID-19 detection may have underestimated the real incidence of infection in our population. Moreover, the sample size may have influenced the significance of some analysis as the difference in mortality in patients treated with ACEi/ARBs or naïve.

## Conclusions

In conclusion, in a high risk population, such as that of patients with STEMI, the potential benefit of ACEi/ARB discontinuation in patients with COVID-19 may be overcome by its detrimental effect. Therefore, our clinical practice should not be modified regarding the treatment of acute ischemic injury among the COVID-19 subpopulation. Strict respiratory monitoring, intensive care and aggressive life support, regardless from swab test result, should be routinely used among patients admitted for STEMI during pandemic.

## Data Availability Statement

The raw data supporting the conclusions of this article will be made available by the authors without undue reservation.

## Ethics Statement

Ethical review and approval was not required for this study with human participants, in accordance with the local legislation and institutional requirements. The patients/participants provided their written informed consent to participate in this study.

## Author Contributions

LB, SC, GS, DT, and GT contributed to conception and design of the study. LB and GT organized the database. GT performed the statistical analysis. LB, GT, DG, and GP wrote sections of the manuscript. All authors contributed to manuscript revision, read, and approved the submitted version.

## Conflict of Interest

The authors declare that the research was conducted in the absence of any commercial or financial relationships that could be construed as a potential conflict of interest.

## Publisher's Note

All claims expressed in this article are solely those of the authors and do not necessarily represent those of their affiliated organizations, or those of the publisher, the editors and the reviewers. Any product that may be evaluated in this article, or claim that may be made by its manufacturer, is not guaranteed or endorsed by the publisher.
